# Metal artifact reduction in spectral computed tomography for intracavity brachytherapy in cervical cancer patients: a prospective study

**DOI:** 10.1186/s13014-025-02767-9

**Published:** 2025-12-01

**Authors:** Yuliang Sun, Yining Chen, Zheng Zeng, Bing Zhou, Haoran Xu, Junfang Yan, Ke Hu, Fuquan Zhang

**Affiliations:** 1 Department of Radiation Oncology, Peking Union Medical College Hospital, Chinese Academy of Medical Sciences & Peking Union Medical College, No. 1 Shuaifuyuan Wangfujing, Dongcheng District, Beijing, 100730 China; 2 Eight-Year Medical Doctor Program, Chinese Academy of Medical Sciences & Peking Union Medical College, Beijing, China; 3 Department of Radiation Oncology, State Key Laboratory of Complex Severe and Rare Diseases, Peking Union Medical College Hospital, Chinese Academy of Medical Science and Peking Union Medical College, Beijing, 100730 China

**Keywords:** Spectral computed tomography, Metal artifact reduction, Cervical cancer, Image-based intracavity brachytherapy

## Abstract

**Background:**

This study aims to evaluate the effectiveness of combining virtual monochromatic images (VMI) with metal artifact reduction (MAR) algorithms in reducing metal artifacts in cervical cancer patients undergoing intracavitary brachytherapy.

**Materials and methods:**

Fifty cervical cancer patients scheduled for intracavitary brachytherapy underwent spectral computed tomography (CT) images. VMI + MAR/no-MAR were reconstructed at energies ranging from 40 to 140 keV. CT attenuation and image noise (standard deviation) were measured to calculate the signal-to-noise ratio (SNR), contrast-to-noise ratio (CNR) for the most prominent artifacts, and the artifact index (AI) for the cervix, bladder, and rectum. Objective metrics were compared between the VMI + MAR and VMI + no-MAR groups. Subjective image quality was evaluated using a 5-point Likert scale, focusing on artifact reduction, soft tissue contrast, and contouring preference. Geometric distortion was assessed by measuring the long axis, short axis, and long-to-short axis ratio (L/S) of the titanium applicator. Contouring consistency was quantified using the Dice Similarity Coefficient (DSC) and 95th percentile Hausdorff Distance (HD95) between two radiation oncologists.

**Results:**

VMI + MAR significantly reduced the AI for the cervix (relative 34.74%-88.51% reduction) and rectum (relative 11.31%-81.35% reduction) compared to VMI + no-MAR across all energies (all *p* < 0.05). SNR increased from 0.70–1.00 to 1.75–1.97, and CNR improved significantly from 90 keV onwards (*p* < 0.05). 140 keV + MAR achieved the highest SNR and CNR with the lowest AI. Subjective assessment further confirmed that 140 keV + MAR offered the best balance between artifact reduction and soft tissue contrast, with significantly higher scores for artifact reduction and contouring preference (*p* < 0.001). Distortion analysis showed that with increasing VMI energy, long (40-140 keV: from 8.10 to 6.17 mm) and short axes (40-140 keV: from 6.93 to 5.55 mm) decreased, and L/S approached 1 (*p* < 0.001). Contouring consistency analysis further demonstrated that 140 keV + MAR achieved the highest DSC and lowest HD95 among all energy-MAR combinations, indicating superior reproducibility of target and organ-at-risk delineations.

**Conclusions:**

For the investigated cases and imaging procedures, high-energy VMI combined with MAR, particularly 140 keV + MAR, provided the most effective balance of artifact reduction, geometric accuracy, and clinical contouring suitability.

**Supplementary Information:**

The online version contains supplementary material available at 10.1186/s13014-025-02767-9.

## Introduction

Cervical cancer is the fourth most commonly diagnosed malignancy among women worldwide [1], with brachytherapy serving as a cornerstone of treatment, particularly for locally advanced disease [2]. Intracavitary brachytherapy, which enables the placement of sealed radioactive sources in close proximity to or within the tumor, delivers a high radiation dose directly to the target while minimizing exposure to surrounding healthy tissues, thereby enhancing therapeutic efficacy [3]. With the advancement of three-dimensional image-guided brachytherapy, magnetic resonance (MR)-guided brachytherapy is now recommended [4, 5]. However, due to practical limitations and economic considerations, computed tomography (CT)-based brachytherapy planning remains more widely adopted in most radiotherapy centers [6–10].

The accuracy of CT-based brachytherapy is often compromised by metal artifacts, which arise from the metallic components used in brachytherapy applicators [11]. These artifacts not only obscure critical anatomical structures but also affect the precision of organ delineation and target contouring [12]. Moreover, while the predominantly used TG-43 algorithm is unaffected [13], model-based dose calculation algorithms such as TG-186, which have been increasingly investigated in brachytherapy and incorporate tissue heterogeneity and electron density, may be adversely influenced by these artifacts [14]. To improve metal artifact reduction, various techniques have been investigated, including the implementation of metal artifact reduction (MAR) algorithms on spectral CT scanner platforms [15]. Effective MAR techniques are essential for improving the reliability of CT-based brachytherapy, ensuring more accurate treatment planning and dosimetric calculations [12].

Furthermore, several previous studies have demonstrated that the MAR algorithm in spectral CT can effectively reduce artifacts caused by metal implants, such as those used in hip or knee arthroplasty, and I-125 seed implantation for mediastinal or hepatic tumors [16–19]. Despite its potential, the application of spectral CT for metal artifact reduction in intracavitary brachytherapy for cervical cancer remains underexplored. The capabilities of spectral CT are particularly valuable in intracavitary brachytherapy, where accurate visualization of the applicator and surrounding tissues is essential for optimal contouring of target and organs at risk (OARs) [20]. While some previous studies have explored methods for reducing metal artifacts in cervical cancer brachytherapy, they have primarily been conducted on phantoms [21, 22]. To the best of our knowledge, no study has yet examined spectral CT for metal artifact reduction in cervical cancer patients. This study aims to investigate the efficacy of spectral CT in reducing metal artifacts for intracavitary brachytherapy in cervical cancer patients.

## Materials and methods

### Patients inclusion

This prospective study was approved by the ethics committee at Peking Union Medical College Hospital (approval no. K5465), and written informed consent was obtained from all patients. The protocol was approved by the clinical trial.gov trial (NCT06433817).

Patients were included who met the following criteria: (1) histological confirmation of cervical cancer, (2) planned intracavity brachytherapy, (3) spectral CT examination (Revolution ES, GE HealthCare) with a standardized protocol (as described in the following), (4) reconstruction of MAR images (as described in the following).

**Fig. 1:**
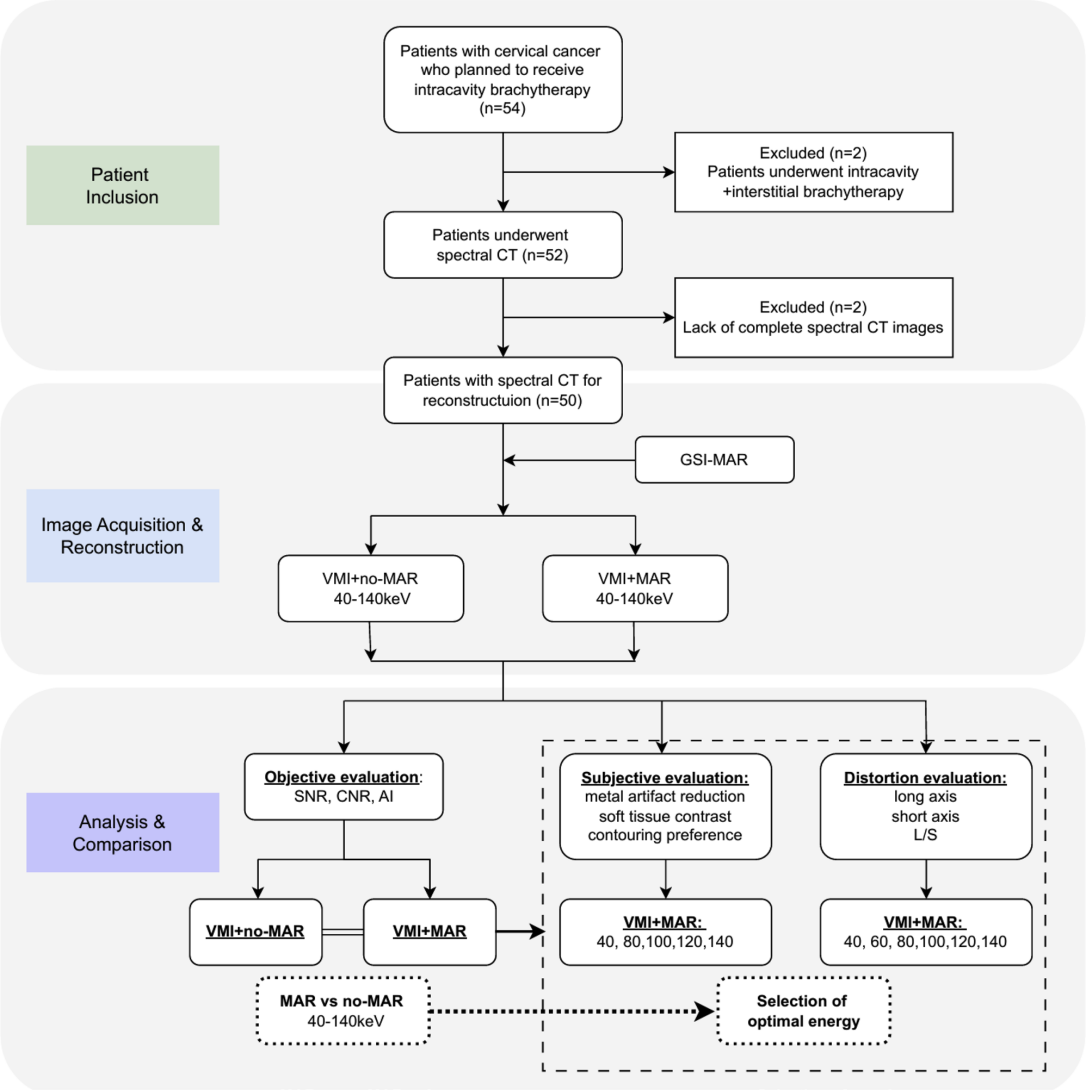
Flowchart for the study. CT, computed tomography; MAR, metal artifact reduction; VMI, virtual monochromatic image; SNR, signal-to-noise ratio; CNR, contrast-to-noise ratio; AI, artifact index; L/S, long-to-short axis ratio

Exclusion criteria were (1) patients who did not undergo intracavitary brachytherapy or received interstitial brachytherapy instead, (2) obvious motion artifacts in the original images, (3) presence of other metal implants in pelvic scanning area, (4) lack of complete spectral images. The flowchart of the experimental design is depicted in Fig. 1. Between October 2023 and October 2024, a total of 50 patients at Peking Union Medical College Hospital were included in the final analysis.

### CT imaging protocols

CT scans for brachytherapy of all patients were performed using fast KV-switching spectral CT, and data analysis was performed on an image post-processing workstation Advantage Workstation, version 4.7 (GE HealthCare, WI, USA). The scanning parameters were as follows: tube current, 480 mA; tube voltage, 80/140 kVp; tube rotation time, 0.8 s/r; pitch, 0.992:1; slice thickness, 5 mm. VMIs with photon energy ranges of 40–140 keV were reconstructed at a 1.25 mm slice thickness using a standard kernel, and both images with MAR algorithm (VMI + MAR) and no-MAR algorithm (VMI + no-MAR) were reconstructed.

### Image quality evaluation

#### Objective evaluation

For each patient, the CT slice demonstrating the most pronounced metal artifact was selected from the reconstructed image sets to assess the effectiveness of artifact reduction. Using 70 keV and 140 keV VMI with MAR, multiple regions of interest (ROIs) were delineated as follows:

Artifact ROI (ROI-A): Placed in the area with the most severe artifact, carefully avoiding intermuscular spaces, bone, and fat to reduce heterogeneity in CT attenuation.

Organ ROIs: Additional ROIs were positioned in artifact-affected regions adjacent to key organs—the cervix (ROI-C), bladder (ROI-B), and rectum (ROI-R).

Muscle ROI (ROI-M): Located in a visually artifact-free muscle area within the same slice to serve as a reference.

Background ROI (ROI-background): Positioned in the air outside the patient’s body, on a slice without metal, to serve as a baseline.

Each ROI measured approximately 15–30 mm² and was drawn three times per patient to minimize measurement variability, with the average values used for analysis. Corresponding ROIs on VMI at other energy levels (both no-MAR and MAR) were replicated using the Gemstone Spectral Imaging software’s duplication function.

For each ROI, the mean CT attenuation and standard deviation (SD) were recorded. Objective image quality metrics—signal-to-noise ratio (SNR), contrast-to-noise ratio (CNR), and artifact index (AI)—were calculated to evaluate soft tissue visualization in artifact-affected regions. SNR and CNR were determined as follows:

$$\scriptsize \:\varvec{S}\varvec{N}\varvec{R}=\frac{1}{3}\sum\limits_{\varvec{i}=1}^{3}\left(\frac{\varvec{m}\varvec{e}\varvec{a}\varvec{n}\:\varvec{R}{\varvec{O}\varvec{I}}_{\varvec{i}}}{{\varvec{S}\varvec{D}}_{\varvec{R}\varvec{O}\varvec{I}\varvec{i}}}\right)$$

$$\scriptsize \begin{aligned}&\varvec{C}\varvec{N}\varvec{R}=\frac{1}{3}\sum\limits_{\varvec{i}=1}^{3}\cr &\left(\frac{|\varvec{m}\varvec{e}\varvec{a}\varvec{n}\:\varvec{R}{\varvec{O}\varvec{I}}_{\varvec{i}}-\varvec{m}\varvec{e}\varvec{a}\varvec{n}\:{\varvec{r}\varvec{e}\varvec{f}}_{\varvec{i}}|}{\sqrt{{\varvec{S}\varvec{D}}_{\varvec{R}\varvec{O}\varvec{I}\varvec{i}}^{2}+{\varvec{S}\varvec{D}}_{\varvec{r}\varvec{e}\varvec{f}}^{2}}}\right)\end{aligned}$$

where mean ROI was the mean CT attenuation of each ROI-A. Therefore, the mean ref, which was the mean CT attenuation of each ROI-M in same slice for the corresponding ROI-A, was subtracted from the respective CT attenuation and SD. And SD_ROI_ and SD_ref_ were their corresponding SD.

AI was used to represent the metal artifacts’ intensity. Since both background noises and metal artifacts could increase the SD of CT attenuation, the influence of background noises (SD_background_) should be subtracted. Then AI was calculated by

$$\small{\varvec{A}\varvec{I}}_{\varvec{x}}=\sqrt{\frac{1}{3}\sum\limits_{\varvec{i}=1}^{3}{\left({\varvec{S}\varvec{D}}_{\varvec{x}\varvec{i}}\right)}^{2}-{\left({\varvec{S}\varvec{D}}_{\varvec{b}\varvec{a}\varvec{c}\varvec{k}\varvec{g}\varvec{r}\varvec{o}\varvec{u}\varvec{n}\varvec{d}}\right)}^{2}}$$

where AI_x_ included AI_cervix_, AI_rectum_ and AI_bladder_. SD_xi_ represented the SD of CT attenuation from ROI-C, ROI-R, ROI-B, and their corresponding AI (AI_cervix_, AI_rectum_ and AI_bladder_) would be calculated respectively. SD_background_ was the mean SD_background_ of SD from the three ROI-background. The SNR, CNR and AI for each patient were calculated using the formula above, then the mean SNR, mean CNR and mean AI would be calculated to compare image quality and metal artifact among VMI of different energies (40–140 keV, 10 keV interval) and MAR or no-MAR.

#### Subjective evaluation

Subjective evaluation was conducted by two radiation oncologists specializing in pelvic tumor imaging, each with over five years of experience in radiological diagnosis, radiotherapy treatment planning, and target contouring. Following the objective assessment of image quality and metal artifacts using quantitative metrics, a subjective evaluation was carried out to identify the optimal VMI energy level with MAR that best fulfills clinical requirements. Based on the objective results, the subjective evaluation focused on VMI + MAR images at five energy levels—40 keV, 80 keV, 100 keV, 120 keV, and 140 keV—as well as on 140 keV VMI + no-MAR. This selection was also guided by the limitation of the 5-point rating scale, which has limited resolution and may not detect subtle differences when too many images are assessed at once. Therefore, the number of images was controlled to ensure that the optimal protocol could still be identified. The reviewers were blinded to the energy levels of the images and were allowed to adjust magnification and window settings as needed. Each image was assessed using a 5-point Likert scale for metal artifact reduction, soft tissue contrast, and contouring preference. The scoring criteria are summarized in Table 1, with contouring preference scored based on relative visual ranking among the six images for the same patient. In cases of discrepancy, the two reviewers reached a consensus through discussion.

**Table 1:** Subjective evaluation criteria of image quality

Score	Metal artifact reduction	Soft tissue contrast	Contouring preference
1	Severe artifact/new artifact	Cannot be distinguished	Not recommended
2	Obvious artifact	Distinguishable but obscured	Slight recommended
3	Moderate	Moderate	Moderate
4	Little artifact	Blurry edged	Highly recommended
5	No artifact	Clear and sharp edged	Same as no artifact

#### Distortion evaluation for MAR

According to previous research results [23], MAR technology effectively mitigates beam hardening artifacts in surrounding tissues but may introduce certain distortions to the metal itself. To evaluate the extent of such distortion, the long and short axes of the titanium catheter within the tandem applicator were measured on VMI + MAR at energy levels of 40 keV, 60 keV, 80 keV, 100 keV, 120 keV, and 140 keV. The axial slice displaying the clearest and most well-defined edges of the titanium tandem was selected for measurement. The longest diameter passing through the center of the catheter was recorded as the long axis, and the shortest diameter through the center was recorded as the short axis. To quantify the degree of shape distortion in the titanium component, the long-to-short axis ratio (L/S) was calculated using the formula: L/S = long axis / short axis. Given that the actual titanium catheter is cylindrical with a diameter of 6 mm on axial sections, the expected L/S ratio is 1.

**Fig. 2:**
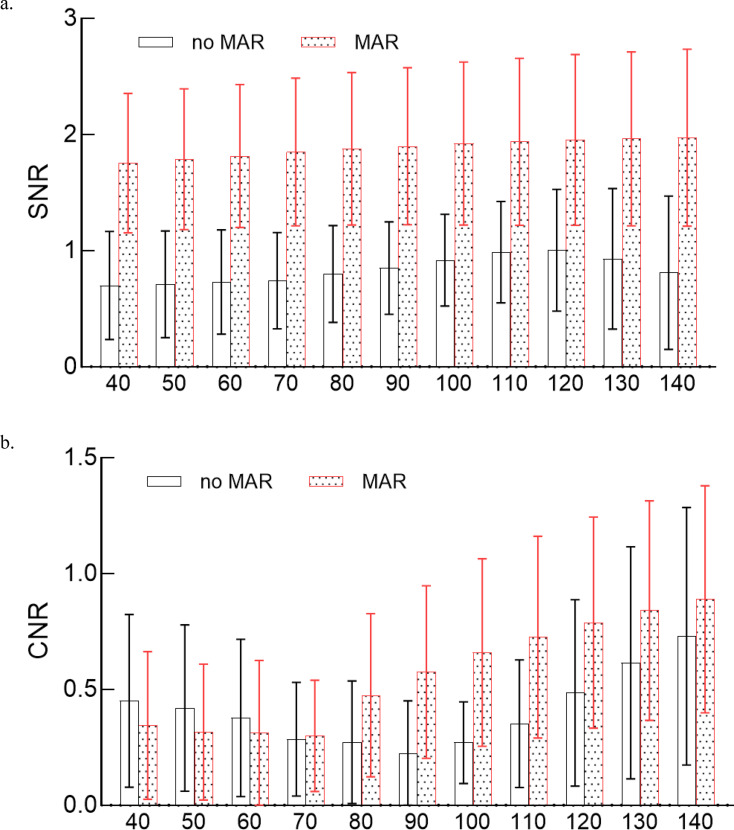
Comparison of SNR and CNR between MAR and no-MAR under different energy. **a**. SNR for MAR and no-MAR of 40–140 keV; **b**. CNR for MAR and no-MAR of 40–140 keV. Data are presented as mean values, and the error bars indicate the standard deviation (SD)

#### Contour consistency evaluation

To evaluate the impact of different VMI energies as well as MAR algorithm on target and OAR contouring consistency, two radiation oncologists with extensive brachytherapy experience independently delineated the high-risk clinical target volume (HR-CTV) and OARs (bladder and rectum) under pelvis window on VMI + MAR images of 40, 80, 100, 120, 140 keV and 140 keV + no-MAR, consistent with the subjective evaluation. Contour consistency was quantitatively assessed using the Dice Similarity Coefficient (DSC) and the 95th percentile Hausdorff Distance (HD95), as commonly reported in radiotherapy imaging studies. DSC was calculated as:

$$\:DSC(A,\:B)=\frac{2\left|A \cap B\right|}{\left|A\right|+\left|B\right|}$$

where *A* and *B* represent the segmented regions in the two contouring of same structure. HD95 was computed as the 95th percentile of the distances from each point on one contour to the nearest point on the other contour, thus reducing sensitivity to outlier voxels. Higher DSC values indicate greater overlap, whereas lower HD95 values indicate better spatial agreement.

### Statistical analysis

All statistical analyses were performed using SPSS version 27.0 (IBM Corp., New York, USA). The normality of data distribution was assessed using the Kolmogorov-Smirnov test. For normally distributed quantitative variables, paired t-tests were used to compare parameters between MAR and no-MAR groups and among different energies. The Mann-Whitney U test was employed to compare subjective evaluation scores. Pearson’s correlation coefficient was calculated to evaluate the strength of association between contouring preference and either metal artifact reduction or soft tissue contrast. Trend analyses were conducted to assess the significance of variation trends in long axis, short axis, and L/S across different VMI energy levels from 40 keV to 140 keV. All the reported percentage increases or decreases of the measured parameters in VMI + MAR were calculated as relative change with respect to the VMI + no-MAR values. A two-sided p-value of < 0.05 was considered statistically significant.

## Result

### Quantitative analysis for objective evaluation

The comparison of SNR and CNR for ROI-A between VMI + MAR and VMI + no-MAR are shown in Table 2. The application of MAR significantly improved the SNR across all 11 VMI energy levels (all *p* < 0.001), with an increase ranging from 0.70–1.00 to 1.75–1.97 (from 95.00% to 152.11%). For VMI + MAR, SNR tended to increase along with the increased energy from 40 keV to 140 keV, shown in Fig. 2a; For VMI + no-MAR, SNR has an increasing trend from 40 keV to 120 keV, but decreased at 130 keV and 140 keV. In total, compared with the SNR of no-MAR images at all energies, MAR images at the same energy had a better SNR, among which 140 keV + MAR has highest SNR.

**Table 2:** SNR and CNR of VMI ± MAR of different energy

VMI energy (keV)	SNR			CNR		
	no MAR	MAR	*p*	no MAR	MAR	*p*
40	0.70 ± 0.47	1.75 ± 0.60	< 0.001	0.45 ± 0.37	0.34 ± 0.32	0.196
50	0.71 ± 0.46	1.79 ± 0.61	< 0.001	0.42 ± 0.36	0.32 ± 0.29	0.288
60	0.73 ± 0.45	1.82 ± 0.62	< 0.001	0.38 ± 0.34	0.31 ± 0.31	0.615
70	0.74 ± 0.41	1.85 ± 0.64	< 0.001	0.29 ± 0.25	0.30 ± 0.24	0.642
80	0.80 ± 0.42	1.88 ± 0.66	< 0.001	0.27 ± 0.26	0.48 ± 0.35	0.017
90	0.85 ± 0.40	1.90 ± 0.68	< 0.001	0.22 ± 0.23	0.58 ± 0.37	< 0.001
100	0.92 ± 0.39	1.92 ± 0.70	< 0.001	0.27 ± 0.18	0.66 ± 0.40	< 0.001
110	0.99 ± 0.44	1.94 ± 0.72	< 0.001	0.35 ± 0.28	0.73 ± 0.44	< 0.001
120	1.00 ± 0.52	1.95 ± 0.74	< 0.001	0.49 ± 0.40	0.79 ± 0.46	< 0.001
130	0.93 ± 0.61	1.96 ± 0.75	< 0.001	0.61 ± 0.50	0.84 ± 0.47	0.004
140	0.81 ± 0.66	1.97 ± 0.76	< 0.001	0.73 ± 0.56	0.89 ± 0.49	0.033

VMI, virtual monochromatic image; SNR, signal-to-noise ratio; CNR, contrast-to-noise ratio; MAR, metal artifact reduction. Data following normal distribution were expressed as mean ± standard deviation

CNR tended to increase with the elevated energy from 70 keV to 140 keV in MAR images, while a decreasing trend could be observed in 40–90 keV for no-MAR images and 40–70 keV for MAR images, as illustrated in Fig. 2b. CNR of VMI + MAR showed significant improvement compared to that of VMI + no-MAR from 90 keV to 140 keV (all *p* < 0.050), whereas there was no statistically significant difference among the MAR and no-MAR pairs of other energies. Among all images of different energy and MAR or no-MAR, 140 keV + MAR had the highest CNR. These results are also presented in Table 2.

**Fig. 3:**
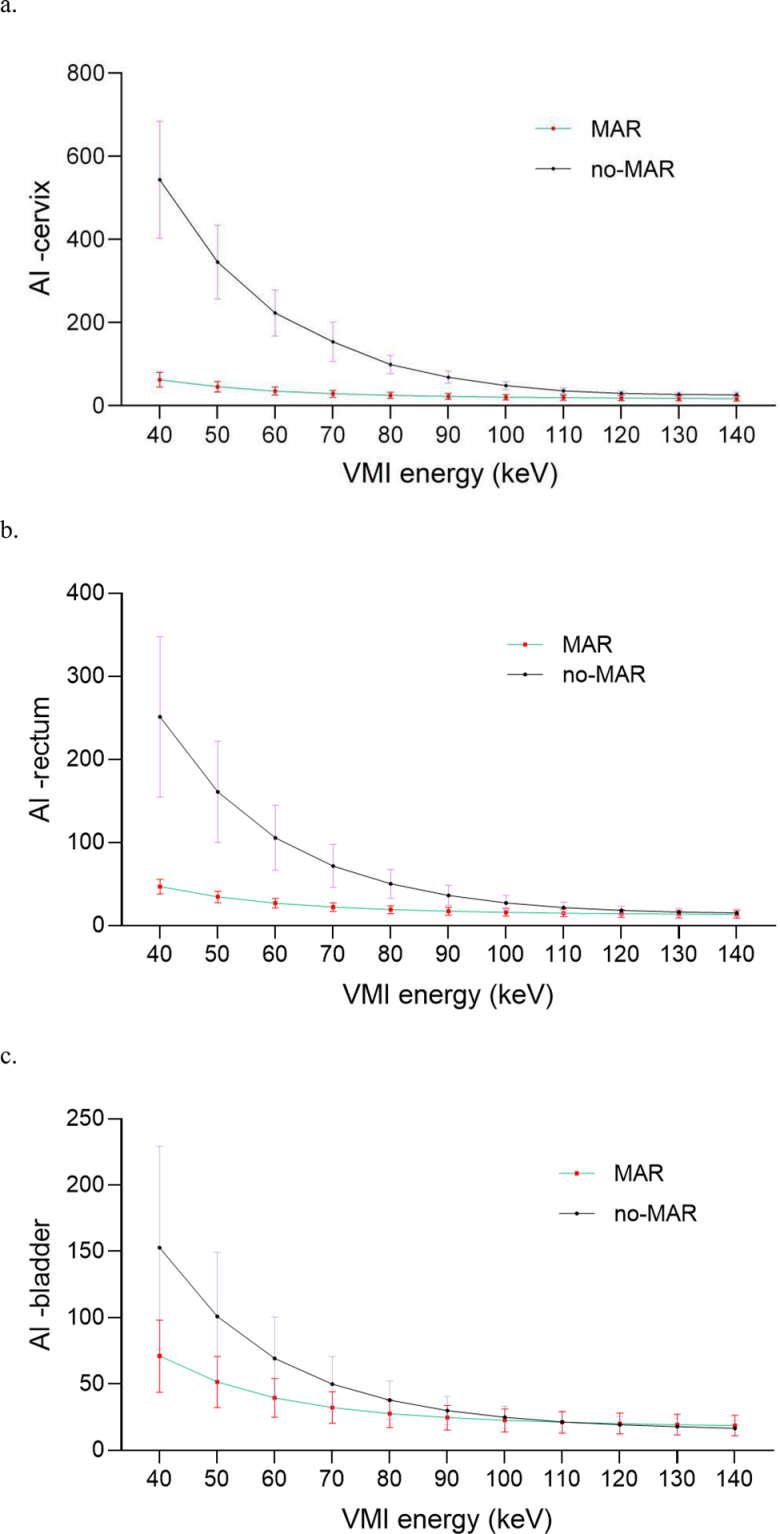
Comparison of AI between MAR and no MAR under different energy. **a**. AI for cervix; **b**. AI for rectum; **c**. AI for bladder. AI, artifact index; MAR, metal artifact reduction; VMI, virtual monochromatic image; MAR

The results of AI are demonstrated in Table 3. It showed that all of the AI-cervix, AI-rectum, and AI-bladder decreased as energy increased, regardless of MAR or no-MAR, which is also illustrated in Fig. 3. For AI-cervix and AI-rectum, VMI + MAR was significantly lower than VMI + no-MAR across all energy levels. For AI-cervix, incorporation of MAR made a significant reduction of 34.74% − 88.51% among 40–140 keV; and for AI- rectum, a reduction of 11.31% − 81.35% were also observed compared to VMI + no-MAR. However, AI-bladder exhibited a little bit different trend. While MAR significantly reduced artifacts compared to no-MAR at 40–100 keV, this effect was less pronounced at higher energy levels. At 120 keV to 140 keV, AI-bladder values with MAR were slightly higher than those with no-MAR, though the difference was not statistically significant.

**Table 3:** AI for bladder, rectum and rectum of different energy

VMI energy (keV)	AI-Bladder			AI-Cervix			AI-Rectum		
	no MAR	MAR	*p*	no MAR	MAR	*p*	no MAR	MAR	*p*
40	152.70 ± 76.57	71.05 ± 27.14	< 0.001	543.70 ± 140.50	62.45 ± 17.77	< 0.001	251.00 ± 96.60	46.81 ± 8.82	< 0.001
50	101.00 ± 48.35	51.52 ± 19.29	< 0.001	345.70 ± 88.47	45.59 ± 12.77	< 0.001	160.90 ± 61.02	34.36 ± 6.80	< 0.001
60	69.33 ± 31.17	39.57 ± 14.67	< 0.001	223.00 ± 55.23	35.30 ± 9.93	< 0.001	105.60 ± 39.24	26.76 ± 5.72	< 0.001
70	49.93 ± 20.74	32.22 ± 11.94	< 0.001	153.80 ± 47.44	28.96 ± 8.35	< 0.001	71.59 ± 25.87	22.06 ± 5.16	< 0.001
80	37.74 ± 14.37	27.60 ± 10.32	< 0.001	99.21 ± 22.03	25.00 ± 7.46	< 0.001	50.02 ± 17.48	19.12 ± 4.83	< 0.001
90	29.99 ± 10.56	24.62 ± 9.30	< 0.001	68.50 ± 14.18	22.43 ± 6.94	< 0.001	36.29 ± 12.20	17.24 ± 4.67	< 0.001
100	24.83 ± 8.30	22.54 ± 8.69	0.041	48.36 ± 9.47	20.64 ± 6.65	< 0.001	27.23 ± 8.80	15.88 ± 4.58	< 0.001
110	21.41 ± 7.03	21.06 ± 8.28	0.421	35.93 ± 6.91	19.38 ± 6.46	< 0.001	21.43 ± 6.64	14.92 ± 4.51	< 0.001
120	19.25 ± 6.36	20.09 ± 8.00	0.920	29.47 ± 5.59	18.52 ± 6.36	< 0.001	18.03 ± 5.30	14.27 ± 4.50	< 0.001
130	17.73 ± 5.99	19.29 ± 7.81	0.292	26.82 ± 5.24	17.84 ± 6.28	< 0.001	16.08 ± 4.55	13.76 ± 4.47	0.01
140	16.66 ± 5.81	18.66 ± 7.66	0.071	26.51 ± 5.82	17.30 ± 6.22	< 0.001	15.03 ± 4.35	13.33 ± 4.46	0.038

AI, artifact index; VMI, virtual monochromatic image; MAR, metal artifact reduction. Data following normal distribution were expressed as mean ± standard deviation

Overall, for AI in the cervix and rectum, 140 keV + MAR provided the optimal results. For the bladder, 140 keV + no-MAR yielded the lowest AI, though it was not significantly different from 140 keV + MAR. Considering all structures collectively, 140 keV + MAR appears to be the optimal setting for artifact reduction.

### Subjective evaluation for clinical preference

**Fig. 4:**
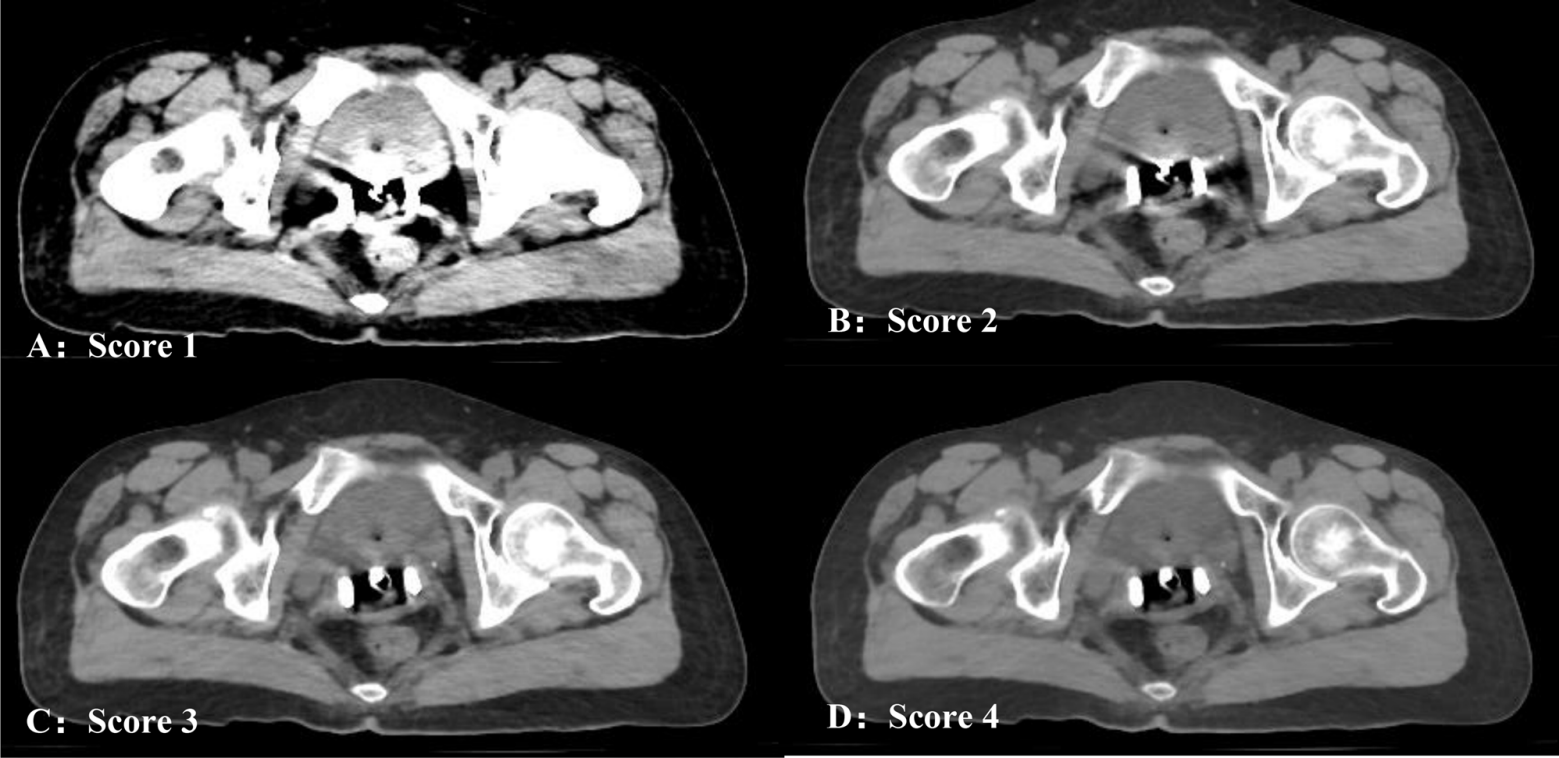
Likert scores of subjective evaluations. This figure used four examples to show the criteria of subjective evaluation. A, B, C and D were the same slice in same patient with different energy, scoring 1, 2, 3 and 4 in both artifact reduction and contouring preference for subjective evaluation in real practice, respectively; for soft tissue contrast, all of the four images could reach score 4

Objective evaluation provided recommendations based on quantitative results, but the clinical feasibility should also be evaluated through oncologists. The scores of subjective evaluations for VMI + MAR of 40 keV, 80 keV, 100 keV, 120 keV and 140 keV, as well as VMI + no-MAR of 140 keV, are shown in Table 4, among which 140 keV had the highest score of metal artifact reduction and contouring preference. For metal artifact reduction and contouring preference, the score increased in VMI + MAR as the energy level increased from 40 keV to 140 keV; meanwhile, 140 keV + MAR had significant higher score than 120 keV + MAR (both *p* < 0.001). As illustrated in Fig. 4, along with the elevation of VMI energy (80 keV, 100 keV, and 140 keV), the metal part of applicator became clearer, and the border of cervix and bladder were better visualized, resulting in a higher score in terms of artifact reduction and contouring preference (score 2, 3, and 4, respectively). And for soft tissue contrast, 40 keV + MAR had the highest score, but only showed limited advantage as its mean score was 4.17 ± 0.38 while the other energies (80–140 keV) scored 4.00 ± 0.00, making slight difference for structure recognition and contouring. Moreover, score 4 for soft tissue contract was good enough for clinical use.

**Table 4:** Scores of subjective evaluations for VMI + MAR of different energy

Energy (keV)	Metal artifact reduction	Soft tissue contrast	Contouring preference
40 + MAR	1.46 ± 0.50	4.17 ± 0.38	1.57 ± 0.49
80 + MAR	2.00 ± 0.00	4.00 ± 0.00	2.00 ± 0.00
100 + MAR	3.00 ± 0.00	4.00 ± 0.00	3.30 ± 0.46
120 + MAR	3.48 ± 0.50	4.00 ± 0.00	3.72 ± 0.45
140 + MAR	4.00 ± 0.00	4.00 ± 0.00	4.00 ± 0.00
140 + no-MAR	3.28 ± 0.45	4.00 ± 0.00	3.46 ± 0.50
P1	< 0.001	> 0.999	< 0.001
P2	< 0.001	> 0.999	< 0.001

P1 shows the significance of scores between 120 keV + MAR and 140 keV + MAR, and P2 shows the significance of scores between 140 keV + MAR and 140 keV + no-MAR. Data were expressed as mean ± standard deviation. VMI, virtual monochromatic image; MAR, metal artifact reduction

The contouring preference was influenced by a combination of metal artifact reduction and soft tissue contrast. Pearson’s correlation analysis revealed a strong positive correlation between metal artifact reduction and contouring preference (Pearson’s correlation coefficient = 0.94, *p* < 0.001), indicating that better artifact reduction was strongly associated with higher contouring preference. In contrast, soft tissue contrast, which would theoretically contribute to contouring, showed a weak negative correlation with contouring preference (Pearson’s correlation coefficient = -0.18, *p* < 0.001), suggesting that its impact was minimal. These findings indicate that metal artifact reduction plays a more dominant role than soft tissue contrast in determining contouring preference.

**Fig. 5:**
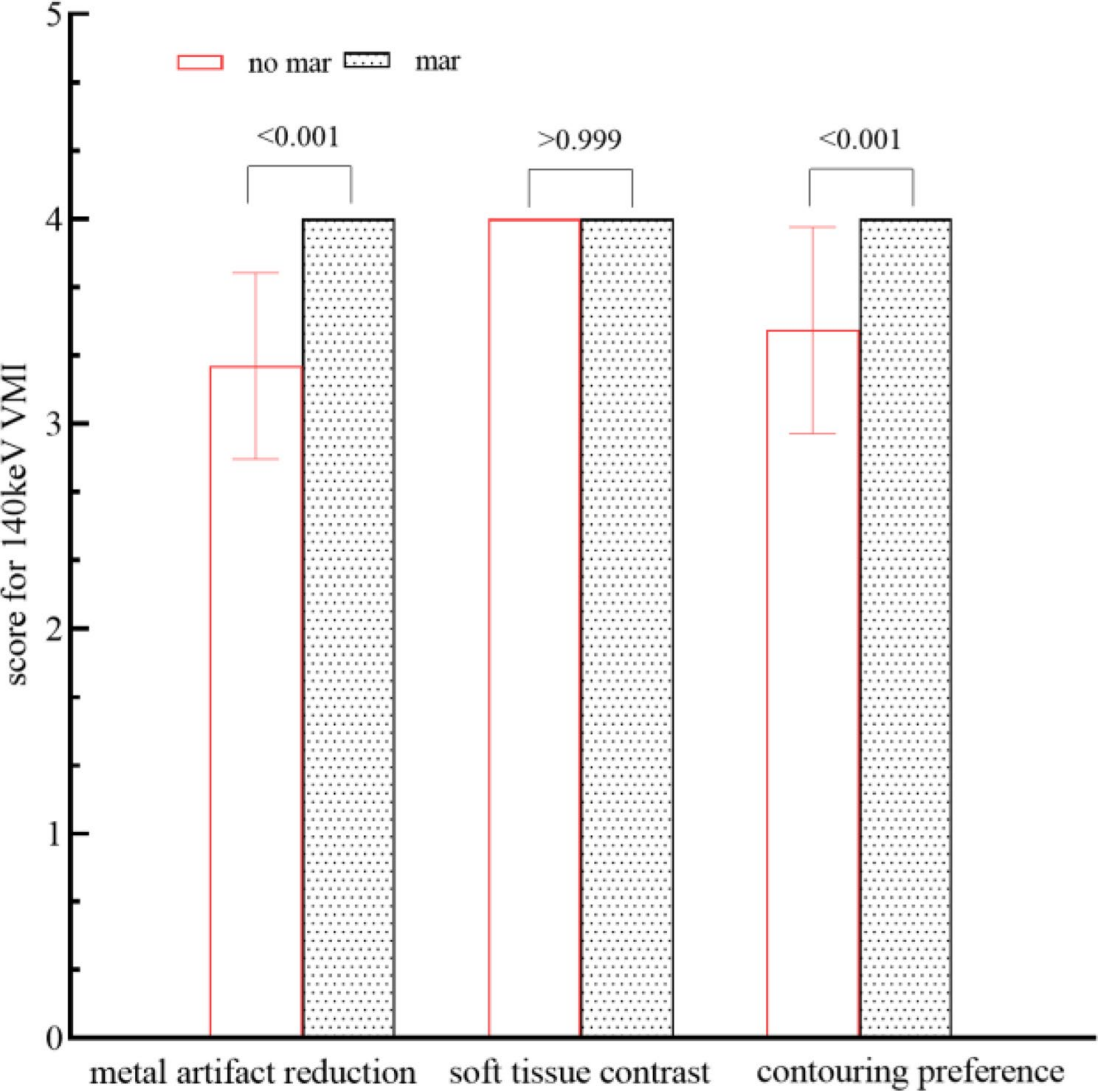
Comparison of 140 keV VMI with and without MAR in subjective evaluation. VMI: Virtual Monochromatic Image; MAR: Metal Artifact Reduction

In addition, a comparison of the subjective evaluations between 140 keV + MAR and 140 keV + no-MAR is presented in Fig. 5. For metal artifact reduction, 140 keV + MAR achieved a significantly higher score than 140 keV + no-MAR (4.00 vs. 3.28, *p* < 0.001). In terms of soft tissue contrast, both 140 keV images—with and without MAR—received a score of 4.00, comparable to the scores observed at 80–120 keV with MAR. For contouring preference, which reflects the combined impact of artifact reduction and soft tissue contrast, 140 keV + MAR also scored significantly higher than 140 keV + no-MAR (4.00 vs. 3.46, *p* < 0.001).

Following the objective evaluation, the results of the subjective assessment further confirmed that 140 keV + MAR not only produced high-quality images with minimal artifacts, but also represented the optimal choice for clinical use by offering the best balance between artifact reduction and adequate soft tissue contrast.

### Distortion evaluation

**Fig. 6:**
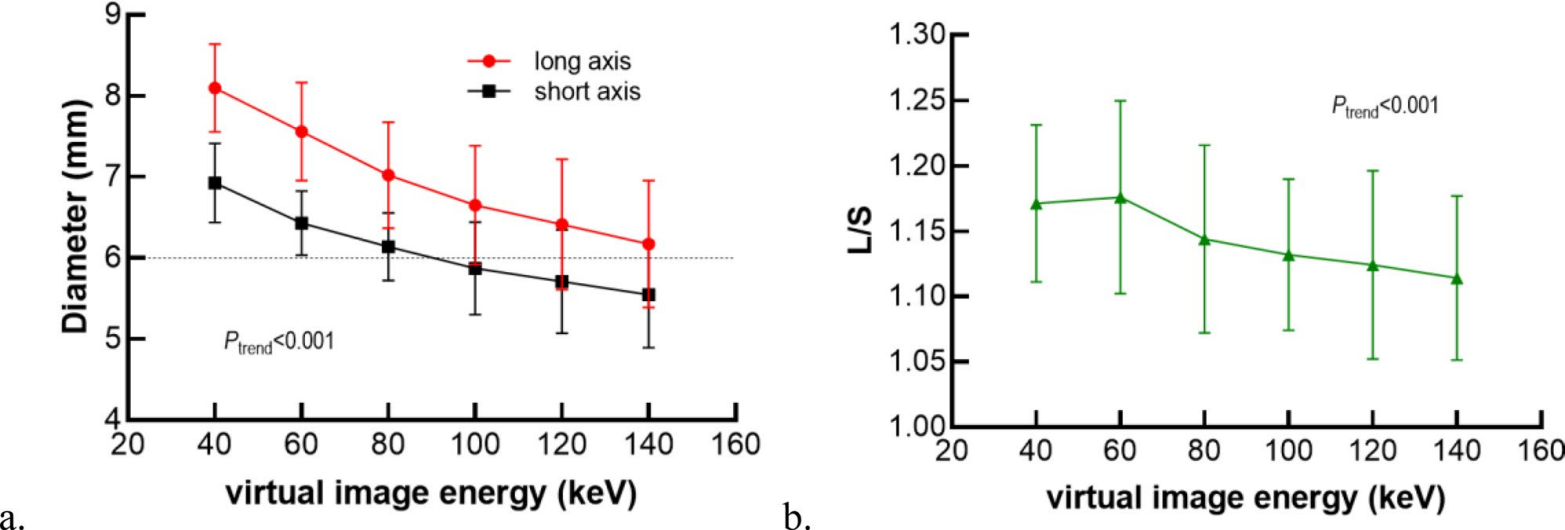
Trend of long axis, short axis and L/S from 40 keV to 140 keV. L/S, long-to-short axis ratio. The dashed line represents the actual diameter value of the measurement

To quantitatively evaluate metal distortion, the titanium component—originally circular with a known diameter of 6 mm—was measured in VMI + MAR images at energy levels ranging from 40 keV to 140 keV in 20 keV increments, as shown in Table 5. A statistically significant decreasing trend was observed in both the long axes (40-140 keV: from 8.10 to 6.17 mm) and short axes (40-140 keV: from 6.93 to 5.55 mm) with increasing energy (*p* < 0.001), along with a gradual reduction in the L/S, which approached 1 at higher energy levels (*p* < 0.001). These decreasing trends in the long axis, short axis, and L/S ratio with increasing VMI energy are illustrated in Fig. 6.

**Table 5:** Measurement result of long axis, short axis and L/S of VMI + MAR

VMI energy (keV)	long axis (mm)	short axis (mm)	L/S
40	8.10 ± 0.54	6.93 ± 0.49	1.17 ± 0.06
60	7.56 ± 0.60	6.43 ± 0.40	1.18 ± 0.07
80	7.02 ± 0.66	6.14 ± 0.42	1.14 ± 0.07
100	6.65 ± 0.74	5.87 ± 0.57	1.13 ± 0.06
120	6.41 ± 0.81	5.71 ± 0.64	1.12 ± 0.07
140	6.17 ± 0.78	5.55 ± 0.66	1.11 ± 0.06
*P*_trend_	< 0.001	< 0.001	< 0.001

Data following normal distribution were expressed as mean ± standard deviation, P_trend_ demonstrated the p value for trend test of long diameter, short diameter and ratio, respectively. L/S, long-to-short axis ratio

### Contour consistency evaluation

Data for DSC and HD95 are presented in Table 6. For HR-CTV and OARs, DSC values for VMI + MAR images showed a slightly but statistically significant increasing trend with increasing energies. All HR-CTV contours achieved DSC values above 0.80, while bladder and rectum contours consistently exceeded 0.95, indicating high baseline agreement. Importantly, while DSC values for bladder and rectum were already close to saturation, HD95 provided a more sensitive measure of boundary deviations. For example, bladder HD95 decreased from 1.797 mm at 40 keV + MAR to 1.158 mm at 140 keV + MAR, and rectum HD95 decreased from 3.089 mm to 2.811 mm across the same energy range. HR-CTV HD95 also showed a decreasing trend, although the increase in 140 keV + MAR compared to 100–120 keV + MAR did not reach statistical significance. The most favorable consistency was observed at 140 keV + MAR, which achieved the highest DSC and lowest HD95 for most structures. Moreover, compared with 140 keV + no-MAR, 140 keV + MAR demonstrated significantly improved agreement for bladder and rectum contours (*p* < 0.001). Figures of a representative case for comparison of the contouring is demonstrated in Supplementary Material 1.

**Table 6:** Contouring consistency of different energy

VMI energy (keV)	DSC			HD95		
	HR-CTV	bladder	rectum	HR-CTV	bladder	rectum
40 + MAR	0.807 ± 0.047*	0.970 ± 0.013*	0.958 ± 0.025*	6.524 ± 1.647*	1.797 ± 0.399*	3.089 ± 0.938*
80 + MAR	0.820 ± 0.045*	0.971 ± 0.012*	0.958 ± 0.025*	6.495 ± 1.609*	1.758 ± 0.347*	3.089 ± 0.937*
100 + MAR	0.821 ± 0.039	0.971 ± 0.010*	0.960 ± 0.027*	6.447 ± 1.573	1.892 ± 0.363*	3.036 ± 0.972*
120 + MAR	0.822 ± 0.039	0.975 ± 0.008*	0.961 ± 0.027*	6.442 ± 1.553	1.667 ± 0.231*	3.036 ± 0.972*
140 + MAR	0.822 ± 0.047	0.984 ± 0.013	0.972 ± 0.017	6.451 ± 1.685	1.158 ± 1.082	2.811 ± 0.657
140 + no-MAR	0.821 ± 0.041	0.972 ± 0.013*	0.960 ± 0.026*	6.452 ± 1.664	1.796 ± 0.398*	3.036 ± 0.972*

HR-CTV, high-risk clinical target volume; VMI, virtual monochromatic images; DSC, Dice Similarity Coefficient; HD95, 95th percentile Hausdorff Distance. Data were expressed as mean ± standard deviation. *: compared with 140 keV + MAR, *p* < 0.001; those values without *: compared with 140 keV + MAR, *p* > 0.05

## Discussion

This prospective study demonstrates that combining VMI at 140 keV with MAR algorithms in spectral CT significantly reduces metal artifacts caused by titanium brachytherapy applicators in cervical cancer patients, while preserving structural fidelity and improving clinical applicability. These findings address a critical challenge in CT-based brachytherapy planning, where metal artifacts compromise contouring accuracy and dose calculation reliability.

Image-guided brachytherapy has become a standard treatment modality for cervical cancer [24]. While MRI-guided brachytherapy offers superior soft-tissue contrast, its limited accessibility and high cost make CT-based planning a necessary alternative in many centers [25]. CT-based brachytherapy remains the primary imaging modality due to its wider availability, faster acquisition times, and seamless integration with treatment planning systems [26]. Meanwhile, in developing regions where cervical cancer remains disproportionately prevalent, resource limitations and overwhelming patient volumes necessitate the continued reliance on metallic tandem-ovoid applicators. Therefore, one of the ongoing challenges is the metal artifacts introduced by applicators, which can compromise the quality of CT images [27]. Apart from the effect on target and OAR delineation for proper contouring, it may also affect dose calculation. Currently, dose calculations in brachytherapy are predominantly performed using the TG-43 algorithm, which assumes a homogeneous water-equivalent medium and does not depend on electron density information. Metal artifacts have limited impact on dose distribution estimates using this approach. However, with the increasing adoption of model-based dose calculation algorithms (such as TG-186) in some centers, which take into account patient heterogeneities and electron density, metal artifacts may potentially affect dose accuracy in these contexts [14, 28, 29]. Therefore, developing an effective and accessible method for reducing metal artifacts in cervical CT images is a critical area of need.

Spectral CT, with its dual-energy capabilities and MAR algorithms, has shown promise in reducing artifacts in orthopedic and dental applications [30]. Multiple studies have indicated that the optimal VMI energy range for reducing artifacts is between 70 and 140 keV, although it is not sufficiently effective in addressing severe metal artifacts [31, 32]. With the integration of MAR algorithms, spectral CT has emerged as a promising solution to enhance material differentiation, reduce artifacts, and contribute to improved vascular structure recognition and tumor visibility [33–35]. Previous studies have demonstrated the effectiveness of combining VMI and MAR to mitigate metal-induced distortions in fields such as orthopedics and CT scanning with dental fillings [36, 37]. Some studies have also highlighted the efficacy of MAR in pelvic organs. Neuhaus et al. found that VMI with MAR provided improved assessment of pelvic organs and adjacent bone compared to VMI alone, although no significant difference was observed in the assessment of adjacent muscles [38]. Similarly, Yue et al. [39] compared monochromatic images with and without MAR in patients undergoing unilateral total hip arthroplasty and found no significant difference for pelvic organs, while visualization of periprosthetic bone, periprosthetic soft tissue, and prosthesis-related problems was improved. Meanwhile, metal artifact reduction can also be applied in the field of radiotherapy, bringing benefits to it.

The use of spectral CT, with its various VMI and MAR algorithms, in mitigating metal artifacts during intracavitary brachytherapy for cervical cancer has not been thoroughly investigated. Kanani et al. [21] compared the effectiveness of the MAR algorithm combined with single-energy CT or dual-energy CT at different energy levels for metal artifact reduction. However, their study only evaluated image quality based on artifact severity and interobserver variability using phantom models. Similarly, Elzibak et al. [22] explored the use of the MAR algorithm for metal artifact reduction in pelvic phantoms during applicator reconstruction, measuring the efficacy through objective indicators such as artifact severity. Both studies were conducted using phantoms and did not report dosimetric results or clinical outcomes.

Our results align with previous phantom-based studies highlighting MAR’s efficacy in reducing artifacts [21, 22], but this is the first clinical validation in patients undergoing intracavitary brachytherapy. Quantitative analysis revealed that 140 keV + MAR achieved the highest SNR and CNR, along with the lowest AI for the cervix and rectum. These improvements are attributed to the suppression of beam-hardening artifacts at higher energy levels and MAR’s ability to correct distorted projections [40]. Notably, subjective evaluations by radiation oncologists confirmed that 140 keV + MAR provided optimal artifact suppression (score: 4.00) without compromising soft-tissue contrast (score: 4.00), making it clinically preferable. The selection of 140 keV as the optimal energy level is supported by both objective and subjective data. Higher keV VMI reduces photon starvation effects and minimizes low-energy noise, which dominates metal artifacts [18, 41]. While lower keV levels (e.g., 40 keV) improved soft-tissue contrast, severe artifacts rendered them clinically impractical. Conversely, 140 keV + MAR balanced artifact reduction with sufficient anatomical visualization, a finding consistent with studies in other anatomical regions [35, 42]. Furthermore, distortion analysis confirmed that 140 keV + MAR maintained the titanium applicator’s geometric integrity (L/S ratio: 1.11), critical for accurate applicator reconstruction [23, 43].

Beyond image quality and geometric distortion, we further evaluated the clinical applicability of artifact reduction by assessing interobserver contouring consistency for subjectively evaluated images. By introducing DSC and HD95, which is the first study, to our knowledge, to evaluate these metrics in metal artifact reduction studies, we quantified contouring reproducibility of target and OAR contours across VMI energies and MAR conditions, thereby directly linking imaging optimization with clinical contouring reproducibility. Importantly, DSC captured the high volumetric overlap (all > 0.8), while HD95 provided additional sensitivity to boundary agreement, highlighting the complementary value of incorporating both metrics. For HR-CTV, the overall DSC remained around 0.82, lower than bladder and rectum, which reflects inherent anatomical challenges in contouring the uterine corpus superiorly and the vaginal margin inferiorly on CT. This variability is consistent with clinical practice and underscores the greater difficulty of target definition compared with OARs. Nevertheless, HD95 still showed a downward trend, suggesting reduced boundary deviations even for the target volume. The 140 keV + MAR protocol not only yielded the highest DSC values and lowest HD95 measurements, but also showed statistically significant improvements over 140 keV + no-MAR images. This indicates that the proposed protocol can directly enhance image quality, thereby reducing interobserver variability and increasing the reliability of target and OAR delineation in brachytherapy planning. Although only modest absolute improvement was observed, the improvement may accumulate over multiple fractions, potentially contributing to long-term clinical benefit.

This study has limitations. First, the energy range was limited to 40–140 keV; higher keV levels (>140 keV) were not explored, though prior studies suggest diminishing returns beyond this range [44]. Second, distortion evaluation focused on the applicator, and potential MAR-induced distortions in adjacent tissues remain unquantified. Additionally, the clinical impact of artifact reduction on dosimetric outcomes warrants further investigation.

Future research should explore MAR’s utility in interstitial brachytherapy, where complex needle arrangements exacerbate artifacts. Comparisons with deep learning-based artifact reduction methods and validation in multi-center cohorts would strengthen clinical relevance [45]. Integrating spectral CT-MAR into treatment planning systems could further enhance brachytherapy precision.

In conclusion, for the investigated cases and available imaging procedures, 140 keV + MAR emerges as the optimal protocol for intracavitary brachytherapy, offering robust artifact reduction, minimal distortion, and enhanced clinical workflow. This approach addresses a longstanding barrier in CT-based brachytherapy, potentially improving treatment accuracy and patient outcomes.

## Conclusions

This study demonstrates that the combined use of MAR and VMI is effective in reducing metal artifacts in intracavitary brachytherapy for cervical cancer. Compared to VMI without MAR, the addition of MAR significantly reduced artifacts caused by titanium applicators. Among all energy levels, VMI + MAR at 140 keV provided the best performance, showing optimal image quality and artifact reduction based on both objective metrics and subjective clinical evaluation. These findings offer practical strategies to improve the accuracy of applicator reconstruction and the delineation of structures affected by metal artifacts in CT-based brachytherapy. By reducing uncertainties in source localization, contouring and dose-volume metrics for both target and normal tissues, this approach has the potential to enhance treatment precision and improve clinical outcomes.

## Supplementary Information

Below is the link to the electronic supplementary material.

Supplementary Material 1

## Data Availability

The datasets used and/or analyzed for the present study are available from the corresponding author on reasonable request.
