# Supplementary material for: Metal artifact reduction in spectral computed tomography for intracavity brachytherapy in cervical cancer patients: a prospective study
Source: Radiat Oncol. 2025 Dec 1;21:3. doi: 10.1186/s13014-025-02767-9 (PMC12777277; doi:10.1186/s13014-025-02767-9)
Supplement: Supplementary file 1 — Supplementary Material 1 [file 13014_2025_2767_MOESM1_ESM.docx]

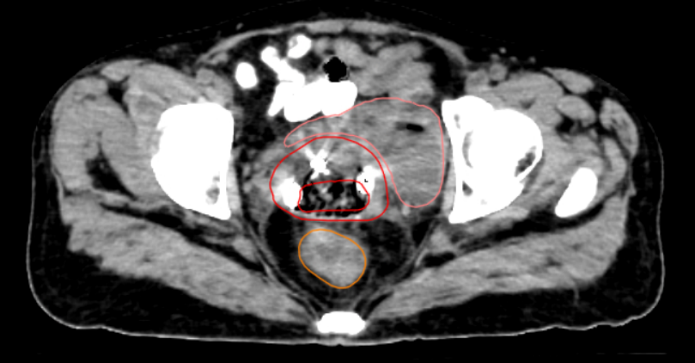

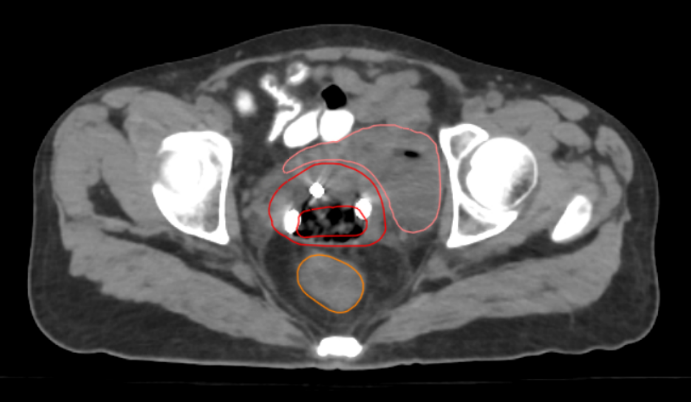


B.

A.


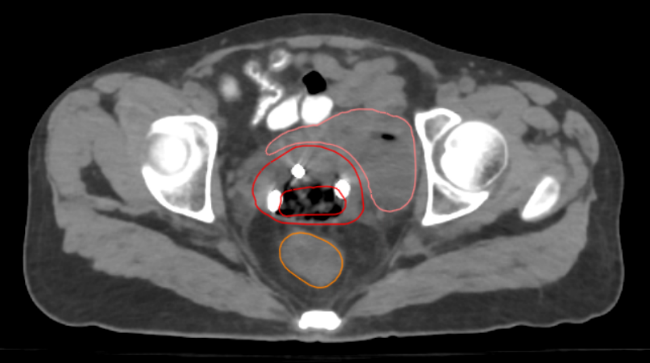

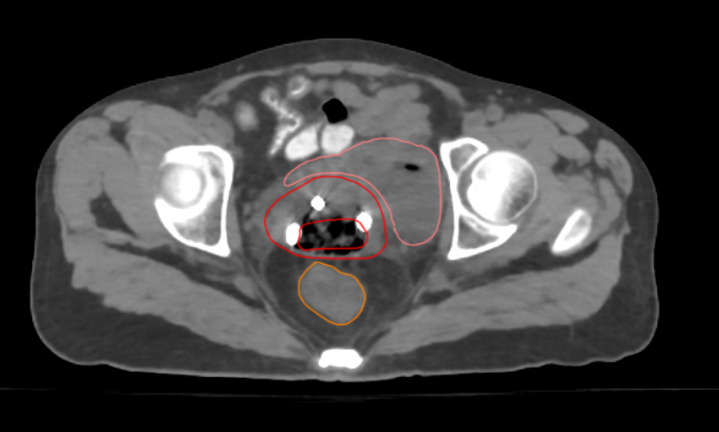


D.

C.


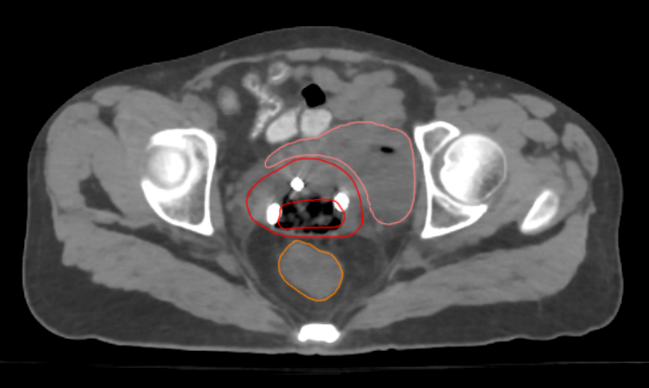

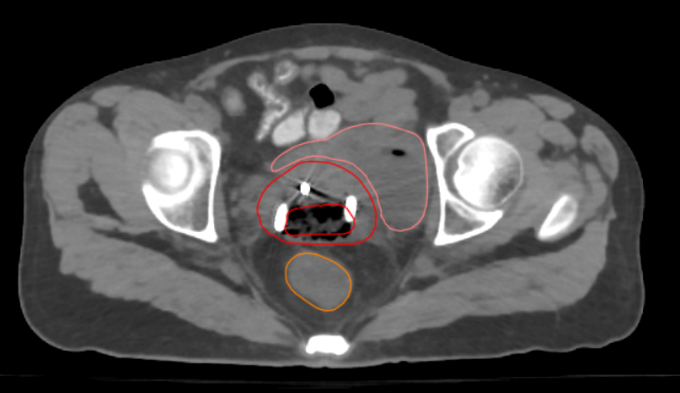


F.

E.

Supplementary File. Representative comparison of contouring among images. All images were under Pelvis window. A. 40 keV+MAR; B. 80 keV+MAR; C. 100 keV+MAR; D. 120 keV+MAR; E. 140 keV+MAR; F. 140 keV+no-MAR.
